# Hollow-core fiber made of ultralow expansion glass: Toward the ultimate stability for room-temperature fiber optics

**DOI:** 10.1126/sciadv.ads7529

**Published:** 2025-06-04

**Authors:** Meng Ding, Ian A. Davidson, Gregory Jasion, Bo Shi, John R. Hayes, Peter C. Schultz, David J. Richardson, Francesco Poletti, Radan Slavík

**Affiliations:** ^1^ Optoelectronics Research Centre, University of Southampton, Southampton SO17 1BJ, UK.; ^2^ Peter Schultz Consulting, 51 Cirillo Dr., Colchester, CT 06415 USA.

## Abstract

Optical fibers have revolutionized many fields including communications, sensing, and manufacturing. Better performance and further applications are expected from emerging hollow-core fibers (HCFs) in which light propagates through a central void. Such propagation eliminates most of the light-glass interaction responsible for most of the transmission impairments in current optical fibers. However, impairments resulting from glass elongation that make the phase and the propagation time of guided light change with external temperature remain even in HCFs. Here, we demonstrate an HCF made from an ultralow expansion glass that exhibits a three orders of magnitude lower coefficient of thermal delay than traditional fibers. This performance, added to the other unique properties of HCFs, opens the door to ultrastable fiber–based applications.

## Teaser

A hollow-core fiber made of ultralow expansion glass shows near-zero thermal sensitivity.

## INTRODUCTION

Single-mode optical fibers (SMFs), because of their many unique properties such as low attenuation, low cost, compactness, and ease of operation, continue to be vital for many important areas of research and technology and attract interest from novel and diverse fields of science and engineering. Besides communications, they are also used in applications such as high-power lasers, fiber-optic gyroscope (*1*), distributed sensing (*2*), distribution of time and frequency reference signals (*3*), and distribution of quantum-encoded signals (*4*). New emerging applications include ultranarrow linewidth lasers stabilized to optical fiber delay lines (*5*), gravitational wave detectors (*6*), interaction between quantum computers (*7*), and many more.

The main performance-limiting impairments of SMFs include attenuation, chromatic dispersion, nonlinearity, and sensitivity of the phase and propagation time of the guided light to environmental changes. These impairments often prevent SMFs from being used in emerging applications or limit their performance in applications where their use is well established. As most of these impairments stem from the light-glass interaction within the SMF glass core, a new class of optical fibers, hollow-core optical fibers made of pure silica glass (silica-HCF) in which light is guided in a hollow, gas- or vacuum-filled core, represents a promising alternative (*8*). Silica-HCFs surpass SMFs in virtually all metrics, yet their fabrication uses near-identical infrastructure, technology, and approach. Recently, HCF design and fabrication have advanced to the point that HCFs with lower attenuation than that achievable in traditional SMFs across the entire optical spectrum from the ultraviolet to mid-infrared have been demonstrated (*9*).

Here, we focus on the sensitivity of the accumulated phase φ and propagation time τ to external temperature (*10*), which, in silica-HCFs, is already 20 to 30 times lower than in SMFs at room temperature. To reduce it even further, we designed and fabricated an HCF using a glass with an ultralow coefficient of thermal expansion (CTE). This fiber, which has the lowest thermal sensitivity to date, has the potential to address the thermal stability requirements of even the most demanding applications and will enable potential all-fiber–based scientific and commercial applications.

**Fig. 1.**
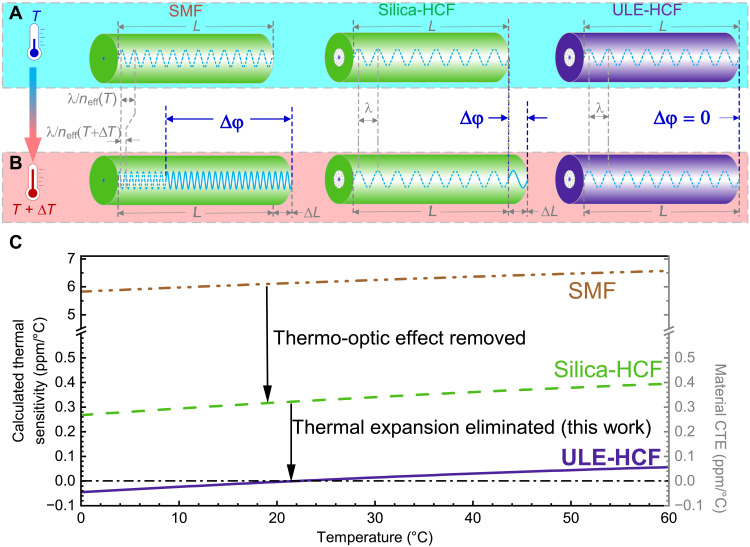
Thermal sensitivity of existing fibers (SMF and silica-HCF) and envisaged HCF made of ultralow expansion glass, ULE-HCF. (**A**) All three fiber types of a length *L* considered at a temperature *T*. (**B**) When the temperature increases by Δ*T*, the thermo-optic effect in the silica glass core through which the light propagates in an SMF (5.8 ppm/°C) is the dominant contributor to the phase change (and, thus, the thermal sensitivity) of an SMF (~6.1 ppm/°C at 20°C) (*33*). In a silica-HCF, the thermo-optic effect is negligible as light travels in air/vacuum, meaning that the phase change is limited by the thermally induced expansion of the silica glass, which is ~0.3 ppm/°C (*33*). By replacing the silica glass with the ULE glass in an HCF, the thermal expansion can be eliminated, providing an optical fiber that is fully thermally insensitive. (**C**) Calculated expected thermal sensitivities of the three considered fiber types using glass (silica or ULE) properties (without considering the coating contribution) over the 0° to 60°C temperature range. The material CTE is shown on the right *y* axis, as silica-HCF and ULE-HCF thermal sensitivities are given by the used material CTE. The zero CTE of the ULE glass can be tuned via composition control (*60*).

In terms of applications requiring thermally ultrastable fibers, interferometers represent one of the key areas. They are the core of many of the most sensitive instruments ever built, dating back to Michelson’s experiment to measure the nonexistence of luminiferous aether to modern day, such as those recently used for gravitational wave detection (*11*), and the stabilization of lasers to 10^−17^ accuracy (*12*). Today, stabilized lasers use free-space interferometers built for lab environments, but making them field deployable and low cost, even at the expense of reduced stability, e.g., 10^−13^ (*13*), would benefit many exciting applications. These include earthquake sensing at the ocean floor (*14*), low-phase noise microwave generation via optical frequency division (*15*), practical quantum physics systems (*16*), high-precision spectroscopy for astronomy (*17*), or multicompound chemical analysis (*18*). Portable designs have been demonstrated via the miniaturization of existing systems (*13*), using whispering gallery modes (*19*), integrated optical microresonators (*20*), or optical fiber delay line interferometers (*21*). However, all these approaches suffer from high alignment sensitivity or long-term instability because of the thermal sensitivity of the material in which the light propagates (*20*), as explained in the case of optical fibers in Fig. 1.

Besides improved long-term performance as discussed above, low thermal sensitivity should also produce low thermoconductive noise, which was reported as the limiting noise factor in strain measurement (*22*) and in the long baseline interferometers used for studying gravitational waves and in transporting fragile quantum states (*6*). Another example is fiber-optic gyroscopes, where the low nonlinearity of silica-HCFs has allowed ultrasensitive fiber–based resonant gyroscopes to be demonstrated (*23*) and where the nonreciprocal thermally induced phase shift (*1*) causes a bias drift of 0.05 degrees/hour (*23*), which needs to be improved below <0.01 degrees/hour for the most demanding applications, like inertial navigation (*24*).

In addition to the thermal stability of the phase of optical signals, the stability of the fiber’s latency [i.e., low or zero thermal coefficient of delay (TCD)] is another key parameter for applications involving time synchronization, for example, in synchrotrons (*25*), linear particle accelerators (*26*), large telescope arrays (*27*, *28*), or phase-arrayed antennae (*29*). Now, the fiber links used for accurate timing are stabilized via round-trip time stabilization, which involves a control system and thus becomes prohibitively complex/unreliable in systems requiring accurate synchronization of many telescopes (*27*, *28*) or lasers (e.g., 100s of lasers for coherent power combination to megawatt power levels or even 1000-by-1000 lasers for future application in propulsion of spacecraft by light). Being able to achieve a timing jitter at the femtosecond level over a kilometer length of fiber would be transformative in these large-scale networks (*30*), requiring a fiber that is 100× less thermally sensitive than those available today (*31*).

Silica-HCF–based systems represent a solution because it guides light through naturally low thermal sensitivity guiding media (air/vacuum) (Fig. 1) with the advantages of fiber, such as alignment stability and the ability to introduce large delays (and, thus, high interferometric sensitivity) in a lightweight, low-cost, and compact form. Unfortunately, the length of an HCF changes with temperature because of the thermal expansion effect, changing the phase that light accumulates when propagating through it. This thermally induced length change is relatively small in silica glass from which existing HCFs are made [0.3 parts per million (ppm)/°C (*32*)], producing more than 20 times smaller optical length change variation than observed in SMFs [~6.1 ppm/°C (*33*)], in which the dominant contributor is the thermo-optic effect in the glass core.

One of the pathways to obtain fiber systems with the required ultralow thermal sensitivity is via reduction of the thermal sensitivity in silica-HCFs. Zero TCD was demonstrated in a silica-HCF, achieved by carefully balancing the thermally induced group velocity changes and fiber elongation (*34*). However, ultralow TCD was only achievable at the edge of the fiber’s optical transmission window, where both chromatic dispersion and attenuation were relatively high. Another approach is to operate silica-HCF at low temperatures, where the CTE of silica crosses zero. For silica-HCF with optimized coating, zero thermal sensitivity was achieved at −134°C (*35*). Although of interest for some applications, low temperatures are impractical in most cases. Zero thermal sensitivity at room temperature was achieved in a silica-HCF coiled on a spool with negative CTE (*36*, *37*), which, while impressive and useful for some applications, is clearly not suitable for any applications where the HCF cannot be coiled, e.g., time synchronization of distant devices (*30*), optical frequency comb distribution (*38*), or distribution of quantum states (*39*). The same limitation applies to another technique (*40*) in which a compensated interferometer is made with a short piece of SMF in the reference arm to compensate for the thermally induced changes of the long silica-HCF arm, which again makes this scheme useful in some cases but does not represent a universal solution.

The ultimate solution is thus to fabricate an HCF from a material with very low or zero CTE (Fig. 1) such as the ultralow expansion (ULE) glass, as we will describe below.

## RESULTS

### ULE-HCF design, fabrication, and characterization

#### Design

Transparent materials with zero CTE include ULE glass from Corning (*41*), Zerodur glass ceramics from Schott (*42*), Clearceram-Z from Ohara (*43*), and ZERØ glass ceramics from Nippon Electric Glass (*44*). Among these, to fabricate the temperature-insensitive HCF in this work, we chose ULE glass as it is a glass (silica glass doped with titania) rather than a glass-ceramic, making it more suited to fiber drawing.

There are several challenges in drawing ULE into a fiber. First, suitable ULE tubes are not available. We obtained these via grinding solid glass billets into cylinders and via subsequent precision drilling and polishing of long holes through them. Various fabrication constraints (length, drilled hole sizes, etc.) limited the dimensions of the achievable tubes. It was therefore essential to reprocess and stretch them using a glass-working lathe producing sizes suitable for HCF fabrication, for which an adapted design was also used. Furthermore, the ULE glass is made via a layer-by-layer deposition technique, resulting in a layered structure that creates internal material stresses that can influence the fiber draw process.

**Fig. 2.**
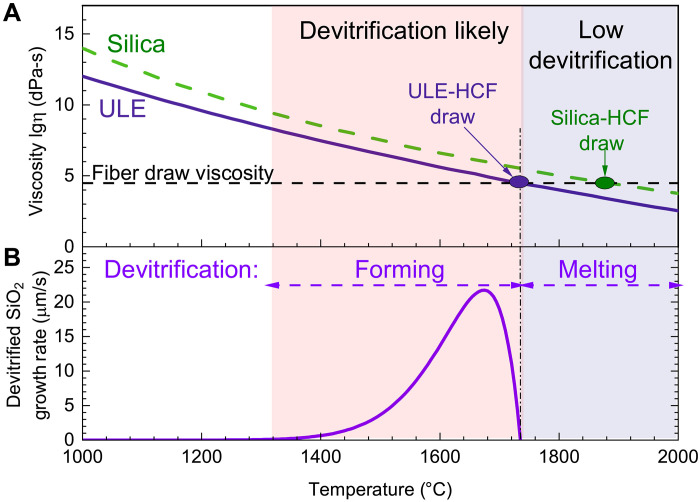
Fiber drawing viscosity and devitrification. (**A**) ULE has lower viscosity than silica, requiring a lower temperature during fiber draw, around 1750°C (ULE) as compared to 1850°C (silica). (**B**) Silica glass devitrification occurs at 1300° to 1728°C, resulting in melting above this temperature, enabling low-devitrification silica fiber drawing within the “low devitrification” region. However, ULE drawing that requires 1750°C is at the very limit of the feasible “low devitrification” draw temperature range (*46*).

Last, a fundamental challenge in drawing this glass into fiber is the danger of causing undesirable glass devitrification, which triggers weak spots and subsequent fiber breaks. The dynamics of glass devitrification is nontrivial; it can be seeded by various crystalline materials and is accelerated by elevated temperatures in the 1200° to 1728°C range (Fig. 2B) (*45*) and by the presence of moisture. However, above ~1728°C, the crystalline devitrified glass (Cristobalite) inclusions very rapidly melt. In a silica fiber draw, any devitrified clusters that might have formed are remelted at the higher draw temperature, because the optimum viscosity for drawing is achieved ~100°C above the maximum crystallization temperature. However, the optimum fiber draw viscosity for ULE glass requires a lower temperature, around 1750°C, reducing the likelihood of remelting of the devitrified clusters. In addition, although less likely to occur because of their low concentration, devitrification of TiO_2_ dopants perhaps does not lead to melting below 1850°C (*46*). To reduce the likelihood of devitrification, in the predraw processing (fire-polishing, etc.), the ULE glass spent the minimum amount of time in the 1200° to 1728°C range. Furthermore, burner ramp rates were selected such that the glass spent substantially longer time at >1728°C than in the 1200° to 1728°C range. This alone should have been sufficient to ensure that the fiber preform is free from devitrified glass. However, to further reduce the likelihood of devitrified glass being present, the fiber was also drawn at a temperature above 1728°C. This was achieved by selecting a peak furnace temperature of >1900°C, which is substantially above 1728°C. This ensured that as the preform entered the ~30-mm-long hot zone (at 4 mm/min), it is rapidly heated to >1728°C because of the furnace being radiation dominated, then spent a brief period of time at the peak temperature (estimated to be >1 s, sufficient to counteract the effective temperature ramp upon entering the hot zone), and was then very rapidly accelerated out of the hot zone to achieve the final line speed of 8 m/min and hence also rapidly cooled to <1200°C.

For our proof-of-concept experiments, we chose a single ring antiresonant (“tubular”) fiber design, which is simpler to produce than modern low-loss HCFs such as nested antiresonant nodeless fibers (*47*). Despite its simplicity, silica-HCFs with these structures have been reported with an attenuation as low as 0.9 dB/km (*48*) at 550 nm and 4.3 dB/km (*49*) at 1000 nm.

We designed a ULE-HCF with seven nontouching tubes (*48*, *49*). This configuration was chosen as it is known to offer the best compromise between supporting robust single-mode guidance (through high attenuation of all high-order modes) and low bend loss. On the basis of simulations considering the size of the available tubes (*50*) and targeted operation around 1550-nm wavelengths, the fiber was designed to have a 46-μm core, 22.5-μm-outer-diameter cladding tubes, ~7-μm intercladding tube gaps, and a cladding tube membrane thickness of 500 nm.

The coatings used to protect fibers can negatively influence the fibers’ overall thermal phase sensitivity, and their effect can be offset by using relatively large glass outer diameters and thin coating thicknesses (see Materials and Methods). In our proof-of-principle demonstration, we targeted a 290-μm ULE-HCF glass diameter with a coating thickness of ~24 μm, although this could be further optimized in future work to further reduce the coating’s influence on the thermal sensitivity, as described in Materials and Methods.

#### Fabrication and characterization

The preform was produced using a standard stack-and-draw method; details are included in Materials and Methods. The fabricated 51-m-long ULE-HCF had a 30-μm-thick acrylate coating, 290-μm outer glass diameter, and 54-μm core size.

**Fig. 3.**
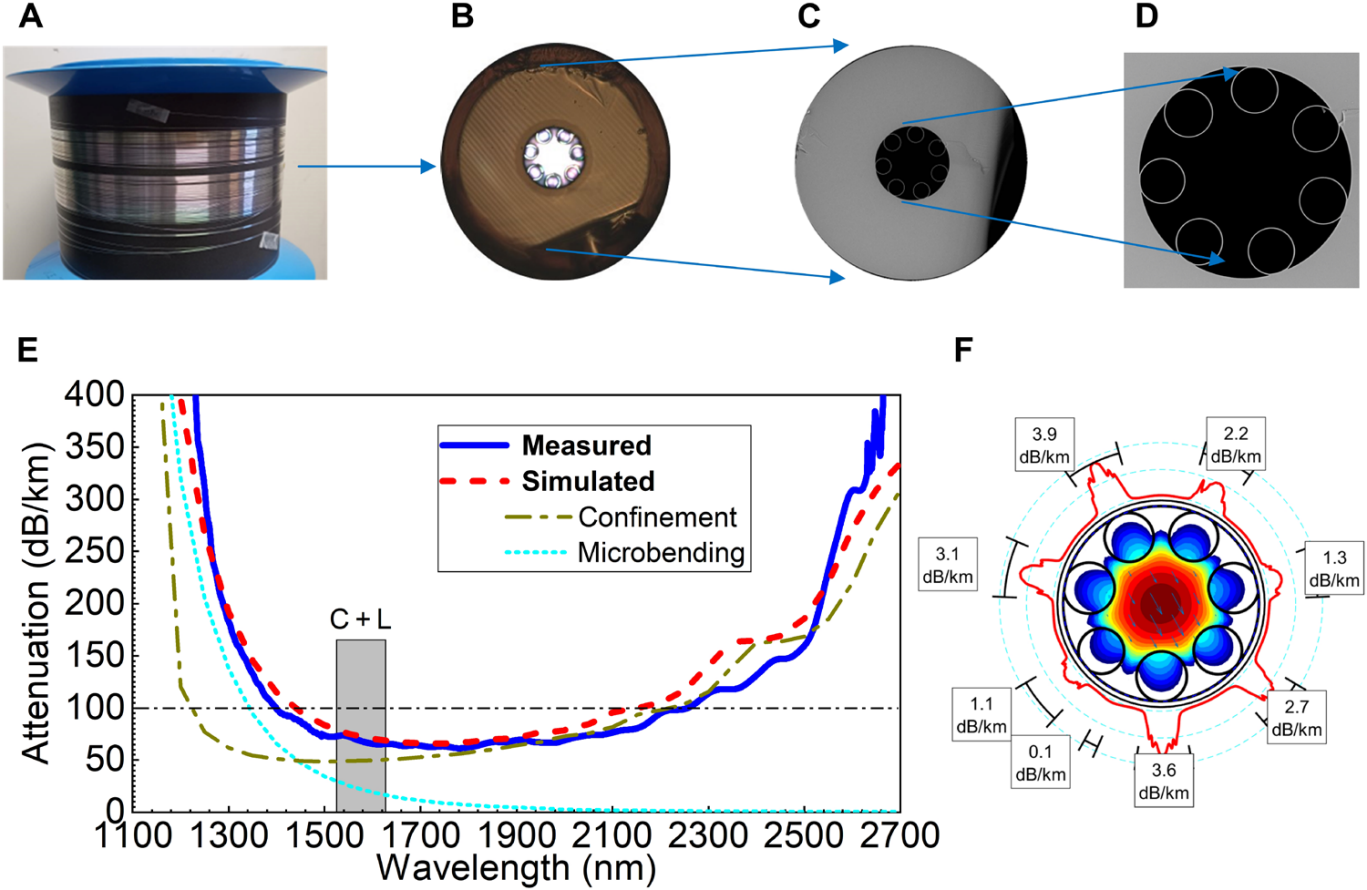
Fabricated ULE-HCF and its attenuation. (**A**) The spooled manufactured fiber shows a pink/green tint resulting from Ti doping (not seen in silica-HCFs). (**B**) Photograph of the end-face showing a thin coating (~30 μm thick) and stripes because of the ULE glass manufacturing process. (**C**) Scanning electron micrograph of the end-face and (**D**) detail of the hollow-core region with a tubular surrounding, showing good ULE-HCF uniformity and symmetry. (**E**) Measured (blue solid) and simulated (red dashes) attenuations. Simulations consider the used bend diameter of 60 cm with its two dominant contributions (confinement, olive green dash-dot; microbending, cyan dot) shown. (**F**) Confinement loss analysis showing the field distribution and leakage loss through the cross section.

Cross-sectional images of simulated and measured attenuations and further analysis are shown in Fig. 3. The attenuation was measured with the cut-back method (see Materials and Methods), achieving a minimum of 70 dB/km at 1790 nm. This agrees well with the simulations in which we considered the coil diameter of 60 cm used during ULE-HCF characterization, suggesting the good optical quality of the manufactured fiber. The two dominant contributors to the attenuation are confinement loss and microbending (*47*), being 50 and 19 dB/km, respectively, at 1550 nm. Although we included material loss in our simulations, estimated at 20 dB/km based on historical fibers from the 1970s with titanium-doped silica cores like ULE glass (*51*), its contribution was found to be negligible. This again confirms the advantage of HCF in which the glass-light overlap is very little. Similarly, surface scattering losses were substantially lower than the confinement and microbending loss in the simulations and are therefore not shown.

The excellent optical performance of the manufactured ULE-HCF despite the nonideal starting material highlights not only successful fabrication but also another major benefit of HCFs, namely their air-core guidance, making material quality a more secondary concern. To the best of our knowledge, the achieved ULE-HCF attenuation of 0.07 dB/m is the lowest loss reported to date for an HCF not made of pure silica glass (*52*–*55*). Because the attenuation is dominated by leakage and microbending, the introduction of nested tubes and optimized structure (*47*) in future design and manufacturing iterations is ultimately expected to yield loss values similar to those of low-loss silica-HCFs, which have been demonstrated to be below 0.1 dB/km recently (*9*).

### ULE-HCF thermal phase sensitivity measurement and TCD evaluation

**Fig. 4.**
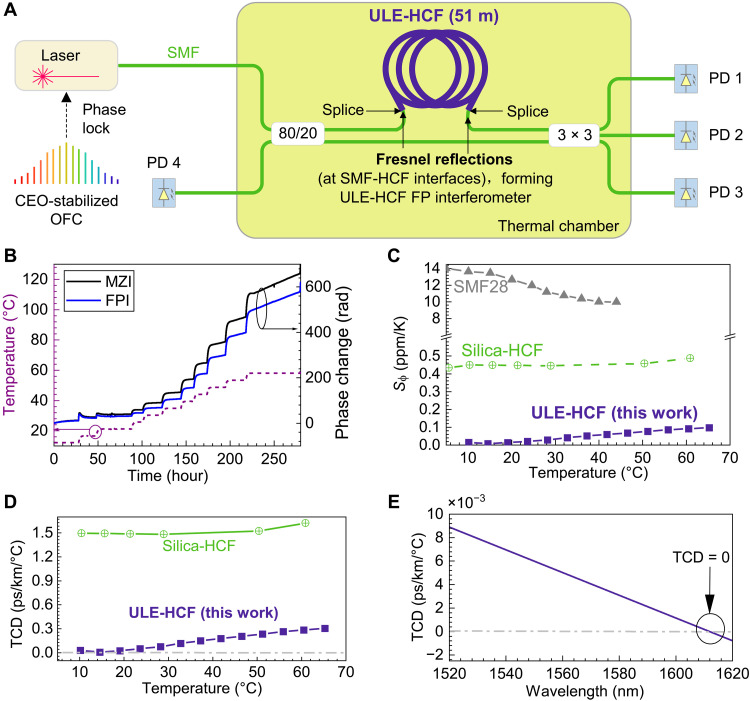
Thermal sensitivity characterization of the ULE-HCF. (**A**) Experimental setup in which the MZI has the ULE-HCF sample in one arm (spliced via mode field adapters to the SMF coupler tails). The SMF length in both arms is matched, strongly suppressing any phase change signal contribution resulting from the SMF. Photodiodes PD 1 to PD 3 monitor the MZI output. Fresnel reflections from the SMFs spliced to the ULE-HCF form an FPI that is monitored with PD 4. CEO-stabilized OFC, carrier envelope offset–stabilized optical frequency comb. (**B**) Measured phase change using MZI (black) and FPI (blue) when the temperature is changed in 5°C steps. (**C**) ULE-HCF thermal phase sensitivity extracted from (B) and its comparison to SMF28 and silica-HCF (*38*). These have slightly different values and temperature dependence as compared to Fig. 1C because of the fiber coating (not considered in the predictions shown in Fig. 1C). (**D**) ULE-HCF TCD at different temperatures. (**E**) ULE-HCF TCD at different wavelengths.

The phase thermal sensitivity of the ULE-HCF was measured using the setup shown in Fig. 4A. The interrogating laser was locked to a carrier envelope offset–stabilized optical frequency comb to eliminate the drift of the interference fringes resulting from laser wavelength drift. The 51-m ULE-HCF sample was loosely coiled with a diameter of 18 cm, which was the maximum diameter we could fit into our thermal chamber, and was spliced into one arm of a Mach-Zehnder interferometer (MZI) that was formed by an 80/20 SMF splitter at the input and a 3-by-3 SMF splitter at the output. The length of the SMFs in both branches was matched to eliminate effects resulting from their thermal sensitivity. The use of a 3-by-3 output coupler enabled phase change extraction including its sign (*32*). The ULE-HCF branch loss was about 7 dB (51 m at 0.07 dB/m and two SMF-HCF splices), which was accommodated by placing it into the 80% MZI branch and by introducing loss in splicing of SMFs in the 20% MZI branch via a lateral offset.

The ULE-HCF sample was spliced to the SMFs of the two MZI couplers using a bridge-fiber mode field adapter (*56*). This created Fresnel reflections at the air-to-glass interfaces at the ULE-HCF input and output, which formed a Fabry-Perot interferometer (FPI) (*36*). In our experiment, we observed both the MZI 3-by-3 output and the FPI reflection via the unused port of the MZI input coupler (Fig. 4A). This combines the advantages of both interferometric methods; specifically, FPI avoids any contribution from the SMFs within the interferometer and gives two times larger phase change, as light goes twice through an FPI. The main advantage of the MZI is that it provides the sign of the phase change.

Subsequently, we removed the ambient pressure and humidity variations (*57*, *58*) by putting the interferometer into a sealed box that we subsequently placed into a thermal chamber. The length-normalized measured accumulated phase change in the FPI and MZI in response to temperature that was changed from 12° to 67°C in 5°C steps is shown in Fig. 4B. At low temperatures, where the coating is relatively stiff (large Young’s modulus), we observe “overshoot” in the phase change following a temperature change, which we attribute to the viscoelastic properties of the coating (*32*). Between 20° and 45°C, this effect is negligible, and the phase change closely follows the temperature change. Above 45°C, we observe a phase drift, which is due to the coating, as we demonstrate in the Supplementary Materials.

The thermal phase sensitivity is then calculated from the FPI signal with removal of the linear thermal phase drift. The normalized thermal phase sensitivity in parts per billion (ppb)/°C is shown in Fig. 4C. The ULE-HCF shows a close-to-zero thermal sensitivity of 8 ppb/°C at 15°C, which is almost 60 times lower than in silica-HCF and 1700 lower than in SMF (*59*) at this temperature (Fig. 4C).

The TCD, which is affected by both phase thermal sensitivity and chromatic dispersion, is hard to measure over the short fiber length used. It can though be evaluated from the phase thermal sensitivity and ULE-HCF chromatic dispersion (see Materials and Methods); details are also included in the Supplementary Materials. The evaluated TCD is shown in Fig. 4D. At 1550 nm, it shows a slightly positive value of 6 fs/km/°C at 15°C, which is 250 times lower than that of the silica-glass HCF and almost 7000 times lower than that of SMF at this temperature. However, as TCD depends on the dispersion that increases with wavelength, TCD = 0 is reached around 1610 nm (Fig. 4E). Within a 100-nm bandwidth, the TCD is below 0.01 ps/km per degree Celsius, which is 4000 times lower than in SMF.

**Fig. 5.**
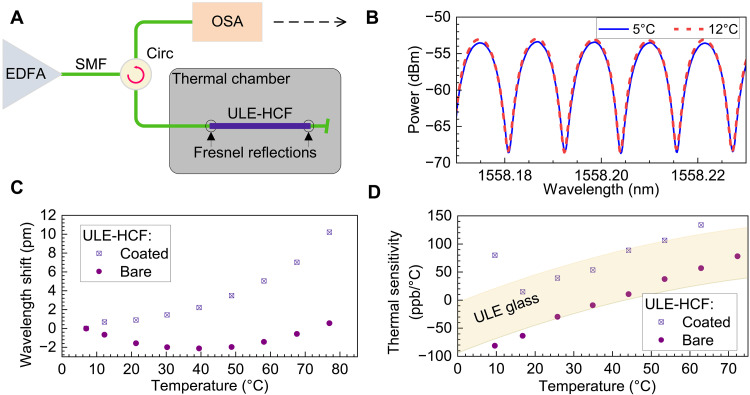
Thermal phase sensitivity characterization of bare ULE-HCF. (**A**) Experimental setup including a shot (~10 cm long)–coated or uncoated ULE-HCF sample spliced with two SMFs to create the FPI (ULE-HCF FPI) via Fresnel reflections at the air-glass (SMF-HCF) interfaces. (**B**) Measured spectral transmission: example using bare ULE-HCF FPI measured at two temperatures. (**C**) Measured wavelength shift of the ULE-HCF FPI for both coated and bare fibers. (**D**) Calculated thermal phase sensitivities from (C). The ULE glass CTE range (ULE7972 from Corning) is shown for comparison as the filled area.

To evaluate the impact of the coating, we used a 10-cm-long piece of coating-stripped ULE-HCF, as longer fiber lengths would be challenging to strip and handle without breaks. We compared it to another 10-cm ULE-HCF sample with the coating intact. We used the FPI method and monitored the transmission directly in the optical domain because the free spectral range of such short FPIs can be resolved by a high-resolution optical spectrum analyzer (Finisar WaveAnalyzer 1500S). We thus replaced the input laser with a broadband light source and analyzed the reflection with the optical spectrum analyzer (Fig. 5A).

The measured interference pattern (Fig. 5B) shifts in wavelength in response to temperature changes made in 7°C steps, as shown in Fig. 5C. From this shift, the ULE-HCF thermal sensitivity was calculated as described in Materials and Methods; the result is shown in Fig. 5D. The bare ULE-HCF’s phase sensitivity is very close to the CTE of ULE: Its range given by the manufacturer is shown as the yellow-shaded area in Fig. 5D. This confirms the finding that the thermal properties of the ULE glass have not changed substantially during the fabrication process, which involved several heating and cooling processes of the ULE glass (extending up to its melting temperature).

## DISCUSSION

We have demonstrated that it is possible to fabricate an optical fiber using an ultralow expansion glass. By using this glass in combination with an HCF design that eliminates the thermo-optic effect from the core of the fiber and by carefully designing the (large) glass diameter and the (thin) coating, we achieved phase thermal sensitivity as low as 0.008 ppm/°C and a TCD of 0.006 ps/km/°C at 1550 nm at room temperature. These are 1700 and 6700 times lower than in SMF, respectively. Zero TCD is achieved at 1610 nm, changing by as little as 10 fs/km/°C over a 100-nm bandwidth, making this fiber suitable for all time delay–sensitive applications. By choosing a ULE glass with lower CTE (using slightly higher Ti doping) and/or by producing a fiber with a different or thinner coating, we are confident that zero phase sensitivity can also be achieved. Besides, although not done here because of the lack of ULE tubes with suitable dimensions, nested tubes will enable a very substantial reduction in the propagation loss of the fiber, making it suitable for operation over long lengths. In any case, with thermal stability >100 times lower than the most thermally insensitive fiber available today, the ULE-HCF demonstrated here already has the required performance to enable many of the applications discussed in Introduction, including the generation of compact and portable ultrapure radio frequency and optical signals with exceptional stability even over >1000-s timescales or practical coherent combination of 1000s of high-power lasers for futuristic applications such as spacecraft light propulsion.

## MATERIALS AND METHODS

### ULE-HCF design and fabrication

The ULE-HCF design was limited by tubes we could source. Silica tubes are made in large volumes by extrusion in a variety of inner/outer diameters to support the worldwide fiber fabrication. For our proof-of-principle experiment, we prepared 50-cm-long tubes from ULE billets by grinding the outer surface into 33- and 36-mm cylinders, respectively. The length was chosen as the maximum length we could get ground into tubes. The diameter was chosen as the maximum diameter that could be processed on our lathe. The maximum was chosen to maximize yield from the tubes. A hole was drilled along the length with diameters of 9.2 and 11.8 mm, respectively, and subsequently polished. Although thinner-wall tubes would be preferred on the basis of the target tubular HCF design, this would increase the likelihood of a break during drilling. The 33-mm ULE tube was then extended on the glass lathe to ~1.5 m, cut, and drawn into tubes on the fiber draw tower, used for manufacturing the HCF cane and both the stack tube and capillaries. The 36-mm ULE tube was extended on the lathe and used as the jacket tube for the canes.

Images of a fabricated cane and fiber can be found in the Supplementary Materials. During this fiber drawing process, tube size was controlled via applying pressures to the seven tubes and to the core area. The wall thickness of cladding tubes was about 400 nm, enabling operation in the first antiresonance window at 1550 nm. The core size was 53 μm. A single-layer acrylate coating was applied with a thickness of 30 μm.

The coatings used to protect fibers negatively increase their thermal phase sensitivity and also introduce a fiber elongation creep following a temperature change resulting from the coating’s viscoelastic properties (*32*). Our previous study shows that a double-coated fiber has higher contribution from the coating as compared to the single-coated one (*24*). Thus, here, we only consider a single-coated HCF. In a single-coated HCF, the simplest model (*32*), for a practical fiber where the coating’s Young modulus is orders of magnitude smaller than that of glass, is

$$S_{\varphi }\approx \alpha_{\text{glass}}+\alpha_{\text{coating}}\frac{E_{\text{coating}}A_{\text{coating}}}{E_{\text{glass}}A_{\text{glass}}}$$ (1)

where E and A are Young’s modulus and area of glass (subscript “glass”) and coating (subscript “coating”), respectively, and α is the CTE of the material. The first term represents the uncoated HCF, while the second term describes the influence of the coating. In ULE-HCF, the second term can easily become dominant, as the first term approaches zero.

To minimize the coating influence, we targeted relatively large *A*_glass_ (290-mm ULE-HCF glass diameter) with relatively small *A*_coating_ (coating thickness of ~24 μm). This coating thickness is larger than a previously demonstrated fiber, which had a coating thickness of 10 μm (*32*), but was selected to ensure the durability of this first proof-of-principle fiber. The used thickness gives $A_{\text{coating}}/A_{\text{glass}}$ of 0.38, which could be reduced in future draws to 0.15 when targeting the 10-μm coating thickness. Further coating optimization can include a coating with smaller Young’s modulus (e.g., acrylate of different compositions) or lower CTE (e.g., Novastart905 polyimide from NeXolve that has CTE close to 0 ppm/°C).

### ULE-HCF attenuation measurement

We coupled a fiberized supercontinuum source (ELECTRO MIR 4.8), which has a spectral bandwidth of 0.9 to 4.8 mm with the average power of 600 mW via SMF into a 7-m-long ULE-HCF using a free-space lens telescope. We then but-coupled it into the ULE-HCF under test (the original 71-m piece unfortunately broke at 51 m) and measured optical power at its input and output to evaluate the attenuation. In this measurement, we neglected the but-coupling loss between two ULE-HCFs, slightly overestimating the attenuation.

### Evaluation of ULE-HCF TCD

The fiber’s thermal sensitivity can be defined in respect of either the phase (thermal phase sensitivity $S_{\varphi }$ ) or the time delay τ (TCD). Thermal phase sensitivity is defined as the phase change normalized by the total phase

$$S_{\varphi }=\frac{1}{\varphi }\frac{d\varphi }{\mathrm{dT}}$$ (2)

where the phase is calculated using the effective index of ULE-HCF

$$\varphi =2\pi \frac{n_{\text{eff}}L}{\lambda }$$ (3)

TCD is defined as the time delay change per unit length (km) per degree Celsius

$$\text{TCD}=\frac{1}{L}\frac{d\tau }{\mathrm{dT}}$$ (4)

where the time delay is calculated using the group index $n_{g}$ of ULE-HCF

$$\tau =\frac{n_{g}L}{c}$$ (5)

The relationship between the group index $n_{g}$ and the effective index $n_{\text{eff}}$ is

$$n_{g}=n_{\text{eff}}-\lambda \frac{{\mathrm{dn}}_{\text{eff}}}{d\lambda }$$ (6)

The relationship between TCD and thermal phase sensitivity $S_{\varphi }$ then can be calculated by

$$\begin{array}{c} \begin{array}{c} \begin{array}{cc} \text{TCD} & =\frac{1}{L}\frac{d}{\mathrm{dT}}\left(\frac{n_{g}L}{c}\right)=\frac{1}{\mathrm{cL}}\frac{d}{\mathrm{dT}}\left[\left(n_{\text{eff}}-\lambda \frac{{\mathrm{dn}}_{\text{eff}}}{d\lambda }\right)L\right]=\frac{1}{2\pi \mathrm{cL}}\frac{d}{\mathrm{dT}}\left(\varphi \lambda -\lambda \frac{d\left(\varphi \lambda \right)}{d\lambda }\right)\\ & =\frac{1}{2\pi \mathrm{cL}}\frac{d}{\mathrm{dT}}\left(\varphi \lambda -\varphi \lambda -\lambda^{2}\frac{d\varphi }{d\lambda }\right)=-\frac{\lambda^{2}}{2\pi \mathrm{cL}}\frac{d^{2}\varphi }{\mathrm{dTd}\lambda }=-\frac{\lambda^{2}}{2\pi \mathrm{cL}}\frac{d}{d\lambda }\left(\frac{d\varphi }{\mathrm{dT}}\right)\\ & =-\frac{\lambda^{2}}{2\pi \mathrm{cL}}\frac{d\left(\varphi S_{\varphi }\right)}{d\lambda }=\frac{n_{\text{eff}}}{c}S_{\varphi }-D\frac{∆\lambda }{∆T}-\frac{\alpha \lambda }{c}\frac{{\mathrm{dn}}_{\text{eff}}}{d\lambda }\\ & =\frac{n_{\text{eff}}}{c}S_{\varphi }-D\frac{∆\lambda }{∆T}-\left(n_{g}-n_{\text{eff}}\right)\frac{\alpha }{c}\approx \frac{n_{\text{eff}}}{c}S_{\varphi }-D\frac{∆\lambda }{∆T} \end{array} \end{array} \end{array}$$ (7)

Here, *D* indicates the chromatic dispersion of ULE-HCF, α refers to the CTE of the ULE-HCF, and $\frac{\Delta \lambda }{\mathrm{\Delta T}}$ refers to the red-shift effect of the transmission window (*34*). It can be evaluated as follows. For a tubular HCF, the resonance wavelength is

$$\lambda_{0}=\frac{2d\sqrt{n^{2}-1}}{m}$$ (8)

where *d* is the thickness of the glass capillaries, *n* is the refractive index of the ULE glass [~1.473 at 1550 nm (*41*)], and *m* is an integer. Differentiating this expression with temperature, we get

$$\frac{1}{\lambda_{0}}\frac{\Delta \lambda }{\mathrm{\Delta T}}=\frac{1}{d}\frac{\mathrm{\Delta d}}{\mathrm{\Delta T}}+\frac{n}{n^{2}-1}\frac{\mathrm{\Delta n}}{\mathrm{\Delta T}}$$ (9)

The first term indicates the CTE of the ULE-HCF, and for our ULE-HCF, it is close to zero. The second term represents contributions from the thermo-optic effect. For the used ULE, the thermo-optic coefficient is 10.7 × 10^−6^/°C (*41*), which leads to

$$\frac{\Delta \lambda }{\mathrm{\Delta T}}=20.8\;\text{pm}/\;{}^{\circ}\;C$$ (10)

Thus, once we know the chromatic dispersion of the ULE-HCF, we can evaluate TCD from the phase thermal sensitivity. More details of chromatic dispersion measurement and results of the ULE-HCF are provided in the Supplementary Materials.

### Thermal phase sensitivity evaluated from the temperature-induced Fabry-Perot resonance wavelength shift

The thermal phase sensitivity in Fig. 5D is calculated by the relative wavelength shift of each dip of the Fabry-Perot signal shown in Fig. 5B

$$S_{\varphi }=\frac{1}{\lambda_{0}}\frac{\Delta \lambda }{\mathrm{\Delta T}}$$ (11)

To understand the relationship between the wavelength shift of the Fabry-Perot signal and thermal phase sensitivity, we calculate them here. The resonance wavelength in an FPI made of HCF at temperatures *T*_0_ and *T*_1_ is

$$\lambda_{m}^{0}=\frac{2n_{\text{eff}}^{0}L_{0}}{m},\lambda_{m}^{1}=\frac{2n_{\text{eff}}^{1}L_{1}}{m}$$ (12)

where *m* is an integer, $n_{\text{eff}}$ is the effective refractive index of the HCF fundamental mode, and *L* is the HCF length. Subscript “0” refers to *T*_0_, and subscript “1” refers to *T*_1_ temperature.

To evaluate the wavelength shift ( $\lambda_{m}^{1}$-$\lambda_{m}^{0}$ ), resulting from the temperature change of *T*_1_-*T*_0_, we need to calculate the effective refractive index and HCF length changes with temperature. The effective refractive index change is due to the thermo-optic effect and chromatic dispersion

$$n_{\text{eff}}\left(\lambda ,T\right)=n_{\text{eff}}^{0}+\frac{{\mathrm{dn}}_{\text{eff}}}{d\lambda }\cdot \Delta \lambda +\frac{{\mathrm{dn}}_{\text{eff}}}{\mathrm{dT}}\cdot \mathrm{\Delta T}$$ (13)

while the thermal expansion is responsible for the length change

$$L_{1}=L_{0}+\alpha L_{0}\cdot \mathrm{\Delta T}$$ (14)

The wavelength shift ( $\lambda_{m}^{1}$-$\lambda_{m}^{0}$ ) is then obtained by subsisting Eqs. 13 and 14 into Eq. 12

$$\begin{array}{c} \begin{array}{c} \;\Delta \lambda =\lambda_{m}^{1}-\lambda_{m}^{0}\\ \approx \lambda_{m}^{0}\cdot \left(\frac{1}{n_{\text{eff}}^{0}}\frac{{\mathrm{dn}}_{\text{eff}}}{d\lambda }\cdot \Delta \lambda +\frac{1}{n_{\text{eff}}^{0}}\frac{{\mathrm{dn}}_{\text{eff}}}{\mathrm{dT}}\cdot \mathrm{\Delta T}+\alpha \cdot \mathrm{\Delta T}\right) \end{array} \end{array}$$ (15)

This can be regrouped to

$$\left(1-\frac{\lambda_{m}^{0}}{n_{\text{eff}}^{0}}\frac{{\mathrm{dn}}_{\text{eff}}}{d\lambda }\right)\cdot \Delta \lambda =\lambda_{m}^{0}\left(\frac{1}{n_{\text{eff}}^{0}}\frac{{\mathrm{dn}}_{\text{eff}}}{\mathrm{dT}}\cdot \mathrm{\Delta T}+\alpha \cdot \mathrm{\Delta T}\right)$$ (16)

which can be rewritten as

$$\frac{\Delta \lambda }{\lambda_{m}^{0}}=\frac{n_{\text{eff}}^{0}}{n_{g}^{0}}\left(\frac{1}{n_{\text{eff}}^{0}}\frac{{\mathrm{dn}}_{\text{eff}}}{\mathrm{dT}}\cdot \mathrm{\Delta T}+\alpha \cdot \mathrm{\Delta T}\right)$$ (17)

Consequently, the normalized wavelength shift and thermal phase sensitivity’s relationship is

$$\frac{1}{\mathrm{\Delta T}}\frac{\Delta \lambda }{\lambda_{m}^{0}}=\frac{n_{\text{eff}}^{0}}{n_{g}^{0}}S_{\phi }\approx S_{\phi }$$ (18)

## Supplementary Materials

### This PDF file includes:

Sections S1 to S3 Figs. S1 to S5 References
