# Supplementary material for: Hollow-core fiber made of ultralow expansion glass: Toward the ultimate stability for room-temperature fiber optics
Source: Sci Adv. 2025 Jun 4;11(23):eads7529. doi: 10.1126/sciadv.ads7529 (PMC12136038; doi:10.1126/sciadv.ads7529)
Supplement: Supplementary file 1 — Sections S1 to S3 Figs. S1 to S5 References [file sciadv.ads7529_sm.pdf]

Science Advances
AAAS

Supplementary Materials for

Hollow-core fiber made of ultralow expansion glass: Toward the ultimate stability for room-temperature fiber optics

Meng Ding *et al.*

Corresponding author: Meng Ding, meng.ding@soton.ac.uk; Radan Slavík, r.slavik@soton.ac.uk

Sci. Adv. **11**, eads7529 (2025)
DOI: 10.1126/sciadv.ads7529

This PDF file includes:

Sections S1 to S3
Figs. S1 to S5
References

Section S1: Images of fabricated cane and fiber

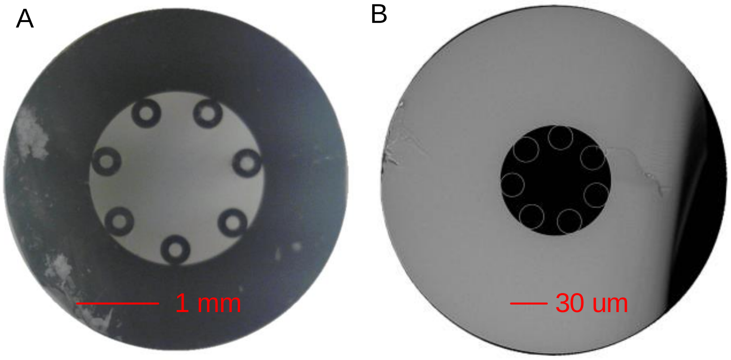

Fig. S1 ULE-HCF. (a) Prepared cane and (b) manufactured ULE-HCF end face.

Section S2: ULE-HCF chromatic dispersion measurement

We used recently-published method (*61*) that uses carrier-envelope-offset (CEO) stabilized optical frequency comb (OFC, FC1500s, MenloSystems). The setup is shown in Fig. S2. OFC has constant tone spacing (repetition rate, f_{rep}), while the ULE-HCF MZI period is frequency dependent due to the chromatic dispersion, Fig. S2. We tuned f_{rep} to be N (N is an integer) times the MZI period at the wavelength of interest. Additionally, we tuned an OFC tone with optical frequency ν_0 to coincide with one of the ULE-HCF MZI transmission peaks. This produces Vernier-like effect, Fig S4. with its period proportional to the ULE-HCF chromatic dispersion.

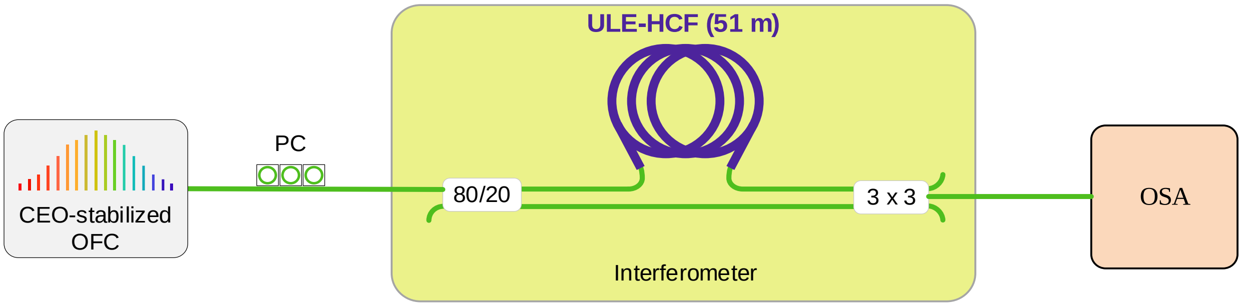

Fig. S2 Measurement setup for ULE-HCF chromatic dispersion. CEO-stabilized OFC: carrier-envelope-stabilized optical frequency comb; PC: polarization controller; OSA: optical spectrum analyzer.

The ULE-HCF length was set to 49.15 m and f_{rep} was tuned to 250.0895 MHz, which corresponds to N = 41. The measured spectrum is shown in Fig. S3 (a). We then calculated the time delay by

$$\delta\tau(\nu) = \frac{1}{N/f_{rep} - f_{rep}^2 / \left(N^2 \cdot FSR_{Envelope}(\nu)\right)}, \tag{S1}$$

where FSR_{Envelope} is the spectral period of the OFC transmitted interferometer obtained from data shown in Fig. S3 (A). The calculated time delay is shown in Fig. S3 (B). We subsequently fitted the time delay curve by the 2nd polynomial and then differentiated it to obtain chromatic dispersion, Fig. S3 (C).

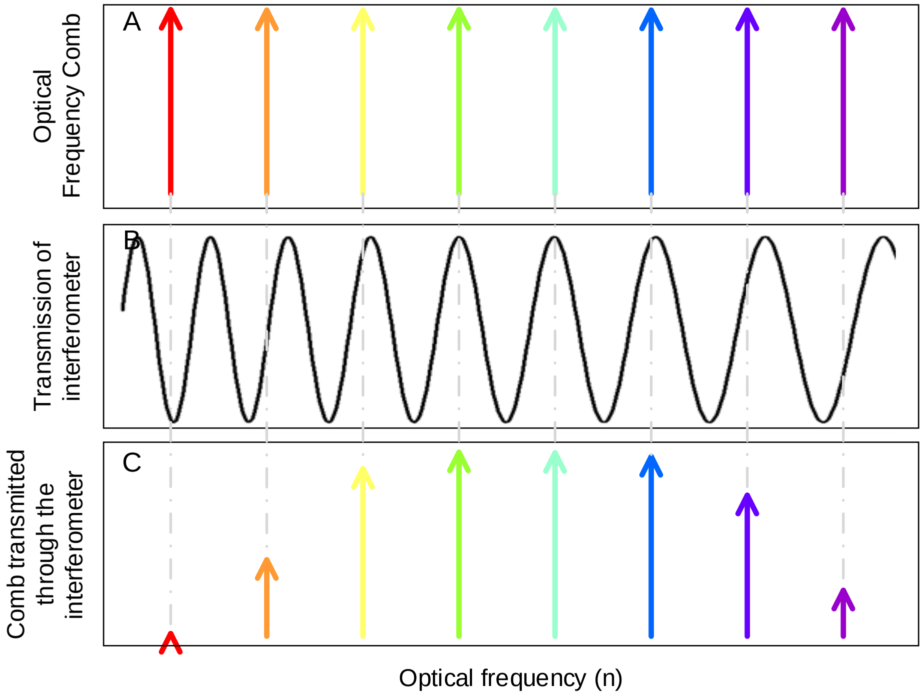

Fig. S3 The principle of used chromatic dispersion measurement method. (A) Optical frequency comb has uniform spacing f_{rep}. **(B)** Spectral period in ULE-HCF interferometer varies due to chromatic dispersion in the ULE-HCF. **(C)** Optical frequency comb transmitted through the interferometer. Based on the envelope of the transmitted power, time delay and chromatic dispersion can be extracted.

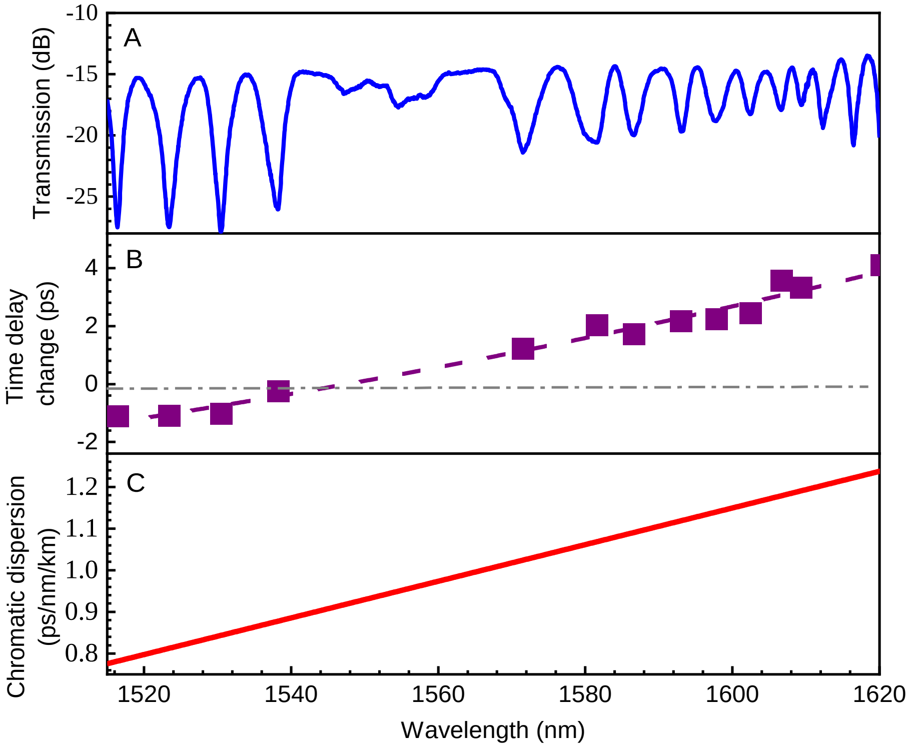

Fig. S4 Measurement of chromatic dispersion. (A) Measured transmitted spectrum of ULE-HCF interferometer using optical frequency comb source. **(B)** Time delay change in the ULE-HCF evaluated from (A). **(C)** Chromatic dispersion evaluated from data in (B).

Section S3: Identification of the coating-induced drift

Fig. S5 shows coated and stripped ULE-HCF FPI wavelength shifts when the temperature was stabilized at 49 ^{o}C for 8 hours. The coated ULE-HCF shows a linear wavelength shift of 8 fm/hour, corresponding to phase change of 0.02 rad/m/hour, which is similar to the phase drift shown in Fig. 5(B). The bare ULE-HCF does not show any drift within our measurement error, suggesting the phase shift shown in Fig. 5(B) is mainly due to the coating.

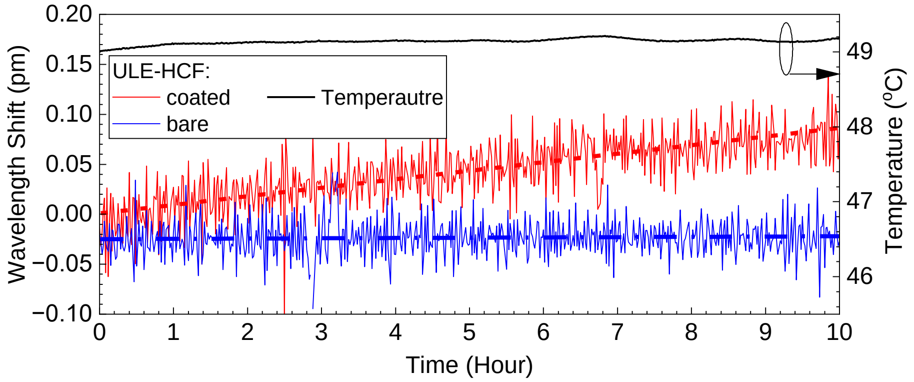

Fig. S5 FPI wavelength drift at constant temperature. Coated (red) and stripped (blue) ULE-HCF FPI wavelength shifts when the temperature was stabilized at 49°C for 8 hours.

REFERENCES AND NOTES

1. D. M. Shupe, Thermally induced nonreciprocity in the fiber-optic interferometer. *Appl. Optics* **19**, 654 (1980).

2. P. Lu, N. Lalam, M. Badar, B. Liu, B. T. Chorpening, M. P. Buric, P. R. Ohodnicki, Distributed optical fiber sensing: Review and perspective. *Appl. Phys. Rev.* **6**, 041302 (2019).

3. K. Predehl, G. Grosche, S. M. F. Raupach, S. Droste, O. Terra, J. Alnis, T. Legero, T. W. Hänsch, T. Udem, R. Holzwarth, H. Schnatz, A 920-kilometer optical fiber link for frequency metrology at the 19th decimal place. *Science* **336**, 441–444 (2012).

4. S. Wengerowsky, S. K. Joshi, F. Steinlechner, H. Hübel, R. Ursin, An entanglement-based wavelength-multiplexed quantum communication network. *Nature* **564**, 225–228 (2018).

5. I. Jeon, C. Ahn, C. Kim, S. Park, W. Jeon, L. Duan, J. Kim, Palm-sized, vibration-insensitive, and vacuum-free all-fiber-photonic module for 10^{-14}-level stabilization of CW lasers and frequency combs. *APL Photonics* **8**, 120804 (2023).

6. C. Hilweg, D. Shadmany, P. Walther, N. Mavalvala, V. Sudhir, Limits and prospects for long-baseline optical fiber interferometry. *Optica* **9**, 1238 (2022).

7. T. Nakamura, T. Nomura, M. Endo, R. He, T. Kashiwazaki, T. Umeki, J. Yoshikawa, A. Furusawa, Low-loss polarization control in fiber systems for quantum computation. *Opt. Express* **31**, 19236 (2023).

8. H. C. H. Mulvad, S. Abokhamis Mousavi, V. Zuba, L. Xu, H. Sakr, T. D. Bradley, J. R. Hayes, G. T. Jasion, E. Numkam Fokoua, A. Taranta, S.-U. Alam, D. J. Richardson, F. Poletti, Kilowatt-average-power single-mode laser light transmission over kilometre-scale hollow-core fibre. *Nat. Photonics* **16**, 448–453 (2022).

9. Y. Chen, M. N. Petrovich, E. Numkam Fokoua, A. I. Adamu, M. R. A. Hassan, H. Sakr, R. Slavík, S. Bakhtiari Gorajoobi, M. Alonso, R. Fatobene Ando, A. Papadimopoulos, T. Varghese, D. Wu, M. Fatobene Ando, K. Wisniowski, S. R. Sandoghchi, G. T. Jasion, D. J.

Richardson, F. Poletti, “Hollow core DNANF optical fiber with <0.11 dB/km loss,” in *Optical Fiber Communications Conference and Exhibition* (Optica Publishing Group, 2024).

10. R. Slavík, G. Marra, E. N. Fokoua, N. Baddela, N. V. Wheeler, M. Petrovich, F. Poletti, D. J. Richardson, Ultralow thermal sensitivity of phase and propagation delay in hollow core optical fibres. *Sci. Rep.* **5**, 15447 (2015).

11. B. P. Abbott, R. Abbott, T. D. Abbott, F. Acernese, K. Ackley, C. Adams, T. Adams, P. Addesso, R. X. Adhikari, LIGO Scientific Collaboration and Virgo Collaboration, GW170817: Observation of gravitational waves from a binary neutron star inspiral. *Phys. Rev. Lett.* **119**, 161101 (2017).

12. D. G. Matei, T. Legero, S. Häfner, C. Grebing, R. Weyrich, W. Zhang, L. Sonderhouse, J. M. Robinson, J. Ye, F. Riehle, U. Sterr, 1.5 µm lasers with sub-10 mHz linewidth. *Phys. Rev. Lett.* **118**, 263202 (2017).

13. M. L. Kelleher, C. A. McLemore, D. Lee, J. Davila-Rodriguez, S. A. Diddams, F. Quinlan, Compact, portable, thermal-noise-limited optical cavity with low acceleration sensitivity. *Opt. Express* **31**, 11954 (2023).

14. G. Marra, C. Clivati, R. Luckett, A. Tampellini, J. Kronjäger, L. Wright, A. Mura, F. Levi, S. Robinson, A. Xuereb, B. Baptie, D. Calonico, Ultrastable laser interferometry for earthquake detection with terrestrial and submarine cables. *Science* **361**, 486–490 (2018).

15. X. Xie, R. Bouchand, D. Nicolodi, M. Giunta, W. Hänsel, M. Lezius, A. Joshi, S. Datta, C. Alexandre, M. Lours, P.-A. Tremblin, G. Santarelli, R. Holzwarth, Y. Le Coq, Photonic microwave signals with zeptosecond-level absolute timing noise. *Nat. Photonics* **11**, 44–47 (2017).

16. I. A. Walmsley, Quantum optics: Science and technology in a new light. *Science* **348**, 525–530 (2015).

17. T. Steinmetz, T. Wilken, C. Araujo-Hauck, R. Holzwarth, T. W. Hänsch, L. Pasquini, A. Manescau, S. D'Odorico, M. T. Murphy, T. Kentischer, W. Schmidt, T. Udem, Laser frequency combs for astronomical observations. *Science* **321**, 1335–1337 (2008).

18. N. Picqué, T. W. Hänsch, Frequency comb spectroscopy. *Nat. Photonics* **13**, 146–157 (2019).

19. C. Panuski, D. Englund, R. Hamerly, Fundamental thermal noise limits for optical microcavities. *Phys. Rev. X* **10**, 041046 (2020).

20. K. Liu, N. Chauhan, J. Wang, A. Isichenko, G. M. Brodnik, P. A. Morton, R. O. Behunin, S. B. Papp, D. J. Blumenthal, 36 Hz integral linewidth laser based on a photonic integrated 4.0 m coil resonator. *Optica* **9**, 770 (2022).

21. F. Kéfélian, H. Jiang, P. Lemonde, G. Santarelli, Ultralow-frequency-noise stabilization of a laser by locking to an optical fiber-delay line. *Opt. Lett.* **34**, 914 (2009).

22. G. Gagliardi, M. Salza, S. Avino, P. Ferraro, P. De Natale, Probing the ultimate limit of fiber-optic strain sensing. *Science* **330**, 1081–1084 (2010).

23. G. A. Sanders, A. A. Taranta, C. Narayanan, E. Numkam Fokoua, S. Abokhamis Mousavi, L. K. Strandjord, M. Smiciklas, T. D. Bradley, J. Hayes, G. T. Jasion, T. Qiu, W. Williams, F. Poletti, D. N. Payne, Hollow-core resonator fiber optic gyroscope using nodeless anti-resonant fiber. *Opt. Lett.* **46**, 46 (2021).

24. V. M. N. Passaro, A. Cuccovillo, L. Vaiani, M. De Carlo, C. E. Campanella, Gyroscope technology and applications: A review in the industrial perspective. *Sensors* **17**, 2284 (2017).

25. F. Loehl, V. Arsov, M. Felber, K. Hacker, B. Lorbeer, F. Ludwig, K. Matthiesen, H. Schlarb, B. Schmidt, A. Winter, S. Schulz, J. Zemella, J. Szewinski, W. Jalmuzna, "Measurement and stabilization of the bunch arrival time at FLASH," in *The 11th European Particle Accelerator Conference (EPAC'08)* (JACoW Publishing, 2008), pp. 3360–3362.

26. M. Xin, K. Şafak, F. X. Kärtner, Ultra-precise timing and synchronization for large-scale scientific instruments. *Optica* **5**, 1564 (2018).

27. SKA, www.skatelescope.org.

28. ALMA, www.almaobservatory.org.

29. E. Nazemosadat, J. I. Herranz-Herruzo, I. Gasulla, Phased array antenna beam-steering in a dispersion-engineered few-mode fiber. *J. Light. Technol.* **41**, 6651–6656 (2023).

30. M. Xin, K. Safak, M. Y. Peng, A. Kalaydzhyan, P. T. Callahan, W. Wang, O. D. Mucke, F. X. Kartner, Breaking the femtosecond barrier in multi-kilometer timing synchronization systems. *IEEE J. Sel. Top. Quantum Electron.* **23**, 97–108 (2017).

31. C. Yan, H. Li, Z. Huang, X. Wang, D. Liu, X. Liu, J. Pan, Z. Luo, F. Yang, Y. Zheng, R. Yin, H. Yu, Y. Leng, L. Song, M. Pang, X. Jiang, Highly stable, flexible delivery of microjoule-level ultrafast pulses in vacuumized anti-resonant hollow-core fibers for active synchronization. *Opt. Lett.* **48**, 1838 (2023).

32. B. Shi, H. Sakr, J. Hayes, X. Wei, E. Numkam Fokoua, M. Ding, Z. Feng, G. Marra, F. Poletti, D. J. Richardson, R. Slavík, Thinly coated hollow core fiber for improved thermal phase-stability performance. *Opt. Lett.* **46**, 5177 (2021).

33. G. Rego, Temperature dependence of the thermo-optic coefficient of SiO_2 glass. *Sensors* **23**, 6023 (2023).

34. E. N. Fokoua, M. N. Petrovich, T. Bradley, F. Poletti, D. J. Richardson, R. Slavík, How to make the propagation time through an optical fiber fully insensitive to temperature variations. *Optica* **4**, 659 (2017).

35. Z. Feng, H. Sakr, J. R. Hayes, E. N. Fokoua, M. Ding, F. Poletti, D. J. Richardson, R. Slavík, Hollow-core fiber with stable propagation delay between −150°C and +60°C. *Opt. Lett.* **48**, 763 (2023).

36. M. Ding, E. N. Fokoua, J. R. Hayes, H. Sakr, P. Horak, F. Poletti, D. J. Richardson, R. Slavík, Hollow-core fiber Fabry–Perot interferometers with reduced sensitivity to temperature. *Opt. Lett.* **47**, 2510 (2022).

37. I. B. Edreira, M. Ding, B. Shi, Z. Feng, G. Marra, I. A. Davidson, J. Rzegocki, S. M. A. Mousavi, G. T. Jasion, F. Poletti, R. Slavík, "Thermal properties of a hollow-core optical fiber spooled onto a drum with negative coefficient of thermal expansion," in *2023 IEEE Photonics Conference (IPC)* (IEEE, 2023), pp. 1–2; https://ieeexplore.ieee.org/document/10360659/.

38. Z. Feng, G. Marra, X. Zhang, E. R. N. Fokoua, H. Sakr, J. R. Hayes, F. Poletti, D. J. Richardson, R. Slavík, Stable optical frequency comb distribution enabled by hollow-core fibers. *Laser Photon. Rev.* **16**, (2022).

39. J.-P. Chen, C. Zhang, Y. Liu, C. Jiang, W.-J. Zhang, Z. Han, S. Ma, X. Hu, Y.-H. Li, H. Liu, F. Zhou, H.-F. Jiang, T.-Y. Chen, H. Li, L.-X. You, Z. Wang, X.-B. Wang, Q. Zhang, J.-W. Pan, Twin-field quantum key distribution over a 511 km optical fibre linking two distant metropolitan areas. *Nat. Photonics* **15**, 570–575 (2021).

40. B. Shi, G. Marra, Z. Feng, H. Sakr, J. R. Hayes, E. R. N. Fokoua, M. Ding, F. Poletti, D. J. Richardson, R. Slavik, Temperature insensitive delay-line fiber interferometer operating at room temperature. *J. Light. Technol.* **40**, 5716–5721 (2022).

41. Corning, ULE Corning Code 7972 Ultra Low Expansion Glass (2016). www.corning.com/media/worldwide/csm/documents/7972%20ULE%20Product%20Information%20Jan%202016.pdf.

42. Schott, Thermal expansion of Zerodur (2010). www.schott.com/en-gb/products/zerodur-p1000269.

43. Oraha, Ultra Low Expansion Glass-Ceramics (CLEARCERAM®-Z). www.oharacorp.com/ccz.html.

44. Nippon Electric Glass Co., ZERØ™(Zero CTE Glass). www.neg.co.jp/en/rd/topics/product-zero/.

45. F. E. Wagstaff, Crystallization and melting kinetics of cristobalite. *J. Am. Ceram. Soc.* **52**, 650–654 (1969).

46. M. Y. Manuputty, C. S. Lindberg, J. A. H. Dreyer, J. Akroyd, J. Edwards, M. Kraft, Understanding the anatase-rutile stability in flame-made TiO_2. *Combust. Flame* **226**, 347–361 (2021).

47. E. Numkam Fokoua, S. Abokhamis Mousavi, G. T. Jasion, D. J. Richardson, F. Poletti, Loss in hollow-core optical fibers: Mechanisms, scaling rules, and limits. *Adv. Opt. Photonics* **15**, 1–85 (2023).

48. J. H. Osório, F. Amrani, F. Delahaye, A. Dhaybi, K. Vasko, F. Melli, F. Giovanardi, D. Vandembroucq, G. Tessier, L. Vincetti, B. Debord, F. Gérôme, F. Benabid, Hollow-core fibers with reduced surface roughness and ultralow loss in the short-wavelength range. *Nat. Commun.* **14**, 1146 (2023).

49. S. Gu, X. Wang, H. Jia, Z. Lian, X. Shen, Y. Mai, S. Lou, Single-ring hollow-core anti-resonant fiber with a record low loss (4.3 dB/km) for high-power laser delivery at 1 μm. *Opt. Lett.* **47**, 5925 (2022).

50. M. Michieletto, J. K. Lyngsø, C. Jakobsen, J. Lægsgaard, O. Bang, T. T. Alkeskjold, Hollow-core fibers for high power pulse delivery. *Opt. Express* **24**, 7103 (2016).

51. R. Won, The birth of optical communications. *Nat. Mater.* **9**, S13–S13 (2010).

52. A. Ventura, J. G. Hayashi, J. Cimek, G. Jasion, P. Janicek, F. Ben Slimen, N. White, Q. Fu, L. Xu, H. Sakr, N. V. Wheeler, D. J. Richardson, F. Poletti, Extruded tellurite antiresonant hollow core fiber for Mid-IR operation. *Opt. Express* **28**, 16542 (2020).

53. X. Jiang, T. G. Euser, A. Abdolvand, F. Babic, F. Tani, N. Y. Joly, J. C. Travers, P. S. J. Russell, Single-mode hollow-core photonic crystal fiber made from soft glass. *Opt. Express* **19**, 15438 (2011).

54. H. Zhang, Y. Chang, Y. Xu, C. Liu, X. Xiao, J. Li, X. Ma, Y. Wang, H. Guo, Design and fabrication of a chalcogenide hollow-core anti-resonant fiber for mid-infrared applications. *Opt. Express* **31**, 7659 (2023).

55. W. Belardi, P. J. Sazio, Borosilicate based hollow-core optical fibers. *Fibers* **7**, 73 (2019).

56. D. Suslov, M. Komanec, E. R. Numkam Fokoua, D. Dousek, A. Zhong, S. Zvánovec, T. D. Bradley, F. Poletti, D. J. Richardson, R. Slavík, Low loss and high performance interconnection between standard single-mode fiber and antiresonant hollow-core fiber. *Sci. Rep.* **11**, 8799 (2021).

57. L. Jin, B.-O. Guan, H. Wei, Sensitivity characteristics of Fabry-Perot pressure sensors based on hollow-core microstructured fibers. *J. Light. Technol.* **31**, 2526–2532 (2013).

58. N. Liu, M. Hu, H. Sun, T. Gang, Z. Yang, Q. Rong, X. Qiao, A fiber-optic refractometer for humidity measurements using an in-fiber Mach–Zehnder interferometer. *Opt. Commun.* **367**, 1–5 (2016).

59. W. Zhu, E. R. Numkam Fokoua, A. A. Taranta, Y. Chen, T. Bradley, M. N. Petrovich, F. Poletti, M. Zhao, D. J. Richardson, R. Slavik, The thermal phase sensitivity of both coated and uncoated standard and hollow core fibers down to cryogenic temperatures. *J. Light. Technol.* **38**, 2477–2484 (2020).

60. K. E. Hrdina, C. A. Duran, ULE® glass with improved thermal properties for EUVL masks and projection optics substrates. *Int. J. Appl. Glas. Sci.* **5**, 82–88 (2014).

61. M. Ding, D. Dousek, A. Zhong, M. Komanec, I. Davidson, G. Jasion, F. Poletti, R. Slavík, “Measurement of chromatic dispersion in hollow core fibers using optical frequency comb,” in *2023 Asia Communications and Photonics Conference/2023 International Photonics and Optoelectronics Meetings (ACP/POEM)* (IEEE, 2023), pp. 1–3.
